# Chemical Profile of Lipophilic Fractions of Different Parts of *Zizyphus lotus* L. by GC-MS and Evaluation of Their Antiproliferative and Antibacterial Activities

**DOI:** 10.3390/molecules27020483

**Published:** 2022-01-13

**Authors:** Sofia Zazouli, Mohammed Chigr, Patrícia A. B. Ramos, Daniela Rosa, Maria M. Castro, Ahmed Jouaiti, Maria F. Duarte, Sónia A. O. Santos, Armando J. D. Silvestre

**Affiliations:** 1 Laboratory of Sustainable Development, Faculty of Science and Technology, University Sultan Moulay Slimane, Beni-Mellal 23000, Morocco; szazouli88@gmail.com (S.Z.); jouaitia@gmail.com (A.J.); 2 Laboratory of Bio-Organic an Analytical Chemistry, Faculty of Science and Technology, University Sultan Moulay Slimane, Beni-Mellal 23000, Morocco; chigrm@gmail.com; 3 CICECO-Aveiro Institute of Materials, Department of Chemistry, Campus de Santiago, University of Aveiro, 3810-193 Aveiro, Portugal; patriciaaramos@ua.pt (P.A.B.R.); armsil@ua.pt (A.J.D.S.); 4 LAQV-REQUIMTE, Department of Chemistry, Campus de Santiago, University of Aveiro, 3810-193 Aveiro, Portugal; 5 Alentejo Biotechnology Center for Agriculture and Agro-Food (CEBAL), Polytechnic Institute of Beja (IPBeja), 7801-908 Beja, Portugal; daniela.rosa@cebal.pt (D.R.); c.mariamiguel@gmail.com (M.M.C.); 6 Mediterranean Institute for Agriculture, Environment and Development—MED, CEBAL, 7081-908 Beja, Portugal

**Keywords:** *Zizyphus lotus* L., lipophilic extracts, gas chromatography–mass spectrometry, pentacyclic triterpenic compounds, antimicrobial activity, triple-negative breast cancer

## Abstract

*Zizyphus lotus* L. is a perennial shrub particularly used in Algerian folk medicine, but little is known concerning the lipophilic compounds in the most frequently used parts, namely, root bark, pulp, leaves and seeds, which are associated with health benefits. In this vein, the lipophilic fractions of these morphological parts of *Z. lotus* from Morocco were studied by gas chromatography–mass spectrometry (GC–MS), and their antiproliferative and antimicrobial activities were evaluated. GC–MS analysis allowed the identification and quantification of 99 lipophilic compounds, including fatty acids, long-chain aliphatic alcohols, pentacyclic triterpenic compounds, sterols, monoglycerides, aromatic compounds and other minor components. Lipophilic extracts of pulp, leaves and seeds were revealed to be mainly composed of fatty acids, representing 54.3–88.6% of the total compounds detected. The leaves and seeds were particularly rich in unsaturated fatty acids, namely, (9*Z*,12*Z*)-octadeca-9,12-dienoic acid (2431 mg kg^−1^ of dry weight) and (9*Z*)-octadec-9-enoic acid (6255 mg kg^−1^ of dry weight). In contrast, root bark contained a high content of pentacyclic triterpenic compounds, particularly betulinic acid, accounting for 9838 mg kg^−1^ of dry weight. Root bark extract showed promising antiproliferative activity against a triple-negative breast cancer cell line, MDA-MB-231, with a half-maximal inhibitory concentration (IC_50_) = 4.23 ± 0.18 µg mL^−1^ of extract. Leaf extract displayed interesting antimicrobial activity against *Escherichia coli*, methicillin-sensitive *Staphylococcus aureus* and *Staphylococcus epidermis*, presenting minimum inhibitory concentration (MIC) values from 1024 to 2048 µg mL^−1^ of extract. Our results demonstrate that *Zizyphus lotus* L. is a source of promising bioactive components, which can be exploited as natural ingredients in pharmaceutical formulations.

## 1. Introduction

*Zizyphus*, a plant genus belonging to the angiosperm Rhamnaceae family, order Rhamnales, includes about 135–170 species worldwide [1], of which *Zizyphus jujuba* Mill. and *Ziziphus mauritiana* Lam. are the most important in terms of distribution and economic significance [2]. *Zizyphus lotus* (*Z. lotus*), also known as “Sedra”, is indigenous to Morocco and has a wide ecological and geographical distribution in arid and semiarid plateau regions and along sandy riverbeds in the Saharan region [3], whereas, in Europe, this plant is restricted to certain semiarid areas, namely, in the southeast of Spain and in Sicily, Italy [4]. This wild shrub is mainly appreciated for its brown, flavorful and nutritive small fruits, which have contributed to the spread of these species. Moreover, *Z. lotus*, particularly its root bark, seeds, fruit pulp and leaves, have been associated with a wide range of health benefits, including in traditional medicine for the treatment of a variety of diseases and disorders, such as liver and urinary complaints, diabetes, skin infections, insomnia, inflammation and peptic ulcers, among others [4,5,6].

An increasing number of studies have identified a variety of bioactive secondary metabolites in *Z. lotus*, particularly phenolic compounds, which have been verified to be present in leaves, branches, and root and stem barks [7,8]. In addition, a vast number of biological properties have been attributed to *Z. lotus* polar extracts from different morphological parts, namely, antioxidant [8,9,10], antibacterial [8], anti-ulcerogenic [10], anti-inflammatory [5,8], analgesic [5], antidiabetic [9] and antispasmodic [11] activities. The less polar fractions of this shrub, however, remain poorly exploited and are most often limited to fatty acid composition [4,6,12,13,14], with a few other compounds, such as seven cyclopeptide alkaloids termed lotusines [15,16,17], four dammarane saponins [18] and one pentacyclic triterpenic compound [19], being previously isolated from leaves, root bark or pulp. However, other interesting families of natural compounds (from a biological activity point of view), such as sterols or tocopherols, have only been evaluated in *Z. lotus* seed oils [12,20] or whole fruit [14] and, in some cases, without quantification [14].

A few biological activities have also been exploited in lipophilic extracts of *Z. lotus*, including antispasmodic [21], anti-ulcerogenic [22], anti-inflammatory and analgesic activities [5]. In this vein, there is a lack of information about the different families of lipophilic compounds present in the different morphological parts of *Z. lotus*, including long-chain aliphatic alcohols, monoglycerides or terpenes. Triterpenic acids have been identified in the genus *Zizyphus* [23,24], but they have not yet been exploited in this shrub species despite their valuable properties, such as antitumor and antiangiogenic activities [25]. The objective of this work is to characterize the lipophilic composition of the morphological parts of *Z. lotus* that are commonly associated with promising biological activities and health benefits, namely, root bark, leaves, seeds and pulp, using gas chromatography–mass spectrometry (GC–MS). The different families of compounds present in these fractions, namely, fatty acids, long-chain aliphatic alcohols, sterols and triterpenic compounds, are qualitatively and quantitatively evaluated here for the first time. In addition, their antiproliferative activity against a triple-negative breast cancer cell line (MDA-MB-231 cells) and their antibacterial activity against *Escherichia coli*, methicillin-sensitive *Staphylococcus aureus* (MSSA) and *Staphylococcus epidermidis* are also evaluated. The characterization and profile comparison of these fractions between morphological parts of *Z. lotus*, together with the evaluation of their respective biological activities, may foster the development of strategies to exploit this shrub for pharmaceutical applications.

## 2. Results and Discussion

### 2.1. Extraction Yield

Dichloromethane (DCM) extracts from the four morphological parts of *Z. lotus* presented very distinct contents of extractives, with seeds showing the highest yield (9.4%), followed by leaves (4.1%), root bark (2.0%) and pulp (1.7%). A similar DCM extraction yield (1.6%) was reported before for *Z. lotus* pulp but was preceded by petroleum ether extraction [26]. DCM lipophilic extractive yields from leaves, seeds and root bark are described herein for the first time.

### 2.2. Lipophilic Composition

**Figure 1:**
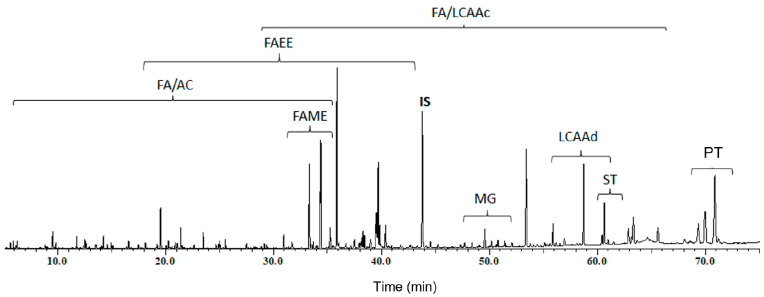
GC−MS chromatogram of the trimethylsilyl-derivatized dichloromethane extract from Z. lotus pulp. Abbreviations: AC, aromatic compounds; IS, internal standard (tetracosane); FA, fatty acids; FAEE, fatty acid ethyl ester; FAME, fatty acid methyl esters; LCAAc, long-chain aliphatic alcohols; LCAAd, long-chain aliphatic aldehydes; MG, monoglycerides; PT, pentacyclic triterpenic compounds; ST, sterols.

The lipophilic fractions of four morphological parts of *Z. lotus*, i.e., root bark, leaves, pulp and seeds obtained by DCM Soxhlet extraction, were characterized in detail using GC–MS analysis. The extract composition is shown in Table 1. As an example, the GC–MS chromatogram of the derivatized DCM extract of pulp is presented in Figure 1.

The GC–MS analysis revealed remarkable diversity of lipophilic constituents of *Z. lotus* extracts, allowing the identification of compounds from six families, namely, fatty acids (including fatty acid ethyl esters (FAEEs) and fatty acid methyl esters (FAMEs)), long-chain aliphatic alcohols, pentacyclic triterpenic compounds, sterols, monoglycerides and aromatic compounds, among other minor compounds.

#### 2.2.1. Fatty Acids

**Figure 2:**
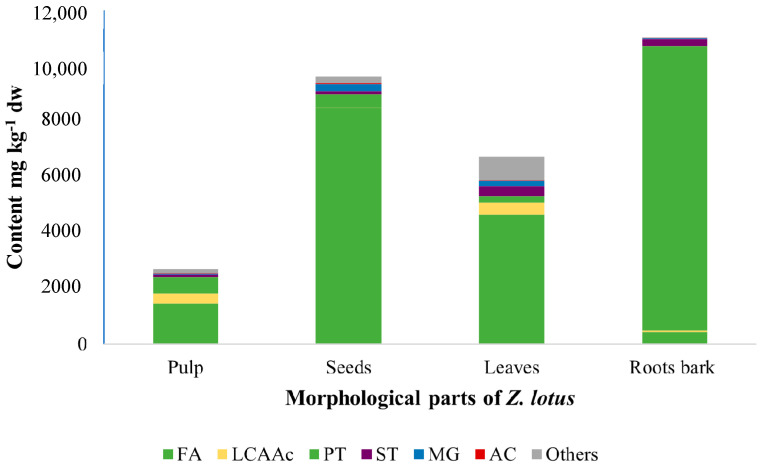
Major families of lipophilic compounds identified by GC–MS in dichloromethane extracts of *Z. lotus*. Abbreviations: AC, aromatic compounds; FA, fatty acids; LCAAc, long-chain aliphatic alcohols; LCAAd, long-chain aliphatic aldehydes; MG, monoglycerides; PT, pentacyclic triterpenic compounds; ST, sterols.

Fatty acids represented the major family of lipophilic components identified in all *Z. lotus* extracts, except for root bark (Table 1 and Figure 2). This family mainly includes saturated and unsaturated fatty acids, a diacid and two *ω*-hydroxy fatty acids that were observed in the four morphological parts of *Z. lotus*. To the best of our knowledge, this family is described for the first time in root bark, while some saturated and unsaturated fatty acids have been previously reported in extracts of leaves, seeds, pulp or whole fruit [4,12,13,14].

A wide diversity of saturated fatty acids (SFAs) (C_10_–C_30_) was identified in this shrub species, with hexadecanoic acid (palmitic acid) as the most abundant SFA in all *Z. lotus* extracts, ranging from 152 mg kg^−1^ in root bark to 877 mg kg^−1^ dry weight (dw) in leaves. Significant amounts of octadecanoic acid were also observed in leaves (276 mg kg^−1^ dw) and seeds (570 mg kg^−1^ dw). Other SFAs were detected in the studied extracts in lower amounts, seven of which (C_21_, C_23_, C_25_–C_28_ and C_30_) were identified for the first time in the present work as *Z. lotus* L. constituents [4,6,12,13,14]. Additionally, to the best of our knowledge, four SFAs in some of the studied morphological parts of *Z. lotus* L. are reported herein for the first time, namely, eicosanoic and docosanoic acids in pulp and leaves and nonadecanoic and tetracosanoic acids in pulp [4,6,12,13].

Unsaturated fatty acids (UFAs) accounted for 28.7–84.8% of the total identified fatty acids of *Z. lotus,* with (9Z)-octadec-9-enoic acid (oleic acid) being the most abundant UFA in *Z. lotus* pulp, root bark and seeds (59–6255 mg kg^−1^ dw). The abundance of UFAs observed in the leaves is mainly due to the presence of (9*Z*,12*Z*)-octadeca-9,12-dienoic (linoleic acid; ω-6) and (9*Z*,12*Z*,15*Z*)-octadeca-9,12,15-trienoic (linolenic acid; ω-3) acids, which accounted for 544 and 2431 mg kg^−1^, respectively. In fact, linolenic acid was the major lipophilic compound detected in leaves, corresponding to 36.1% of all identified compounds in this fraction. Regarding *Z. lotus* pulp, to the best of our knowledge, six of the UFAs detected in this study are novel components of this fraction, namely, tetradecenoic acid, three heptadecenoic acid isomers, (9*Z*,12*Z*,15*Z*)-octadecatri-9,12,15-enoic and eicos-11-enoic acids [4,12,13,14]. Hexadecenoic and heptadecenoic acids were found to be present in three positional isomer forms each; however, their exact configurations were not possible to determine.

Omega-3 and ω-6 polyunsaturated fatty acids (PUFAs) are known to be essential fatty acids in the human diet. Both types offer health benefits; however, the importance of a balanced intake of ω-6 and ω-3 PUFAs is necessary to prevent and manage many diseases [27]. The ideal ω-6/ω-3 ratio is between 1 and 5, which has been related to a significant decrease in inflammatory, cancer, cardiovascular and autoimmune diseases [27]. In the case of *Z. lotus* pulp extract, this ratio, which corresponds to the linoleic/linolenic acid ratio, is approximately 1.33, highlighting the potential of *Z. lotus* pulp to be exploited in nutraceutical applications. Moreover, minor amounts of (9*E*)-otadec-9-enoic acid (18–135 mg kg^−1^ dw) and eicos-11-enoic acid (<0.5–66 mg kg^−1^ dw) were also detected in the four morphological parts studied.

A diacid, namely, hexadecanedioic acid, was detected among the minor components in *Z. lotus* L. pulp lipophilic extracts. Additionally, two *ω*-hydroxy fatty acids were mainly found in the pulp fraction, as shown in Table 1, with a value of 10 mg kg^−1^ dw.

Finally, a wide range of fatty acid esters were identified predominantly as components of *Z. lotus* pulp, with ethyl hexadecanoate as the major component of this subfamily, accounting for up to 44.8% of the total FAEE content identified in the extract of this fraction (Table 1). Minor amounts of ethyl hexadecanoate and ethyl (9*Z*)-octadec-9-enoate were also detected in seeds, accounting for 5 and 11 mg kg^−1^ dw, respectively. Other fatty acid esters, namely, ethyl decanoate and ethyl eicosanoate, were also detected, although in considerably lower amounts (Table 1). Ethyl hexadec-9-enoate and ethyl octadec-9-enoate were both found in *cis* and *trans* configurations, but it was not possible to unambiguously differentiate between stereoisomers. Two fatty acid methyl esters were also identified, namely, methyl (9*Z*)-octadec-9-enoate detected in seeds and root bark extracts and methyl hexadecanoate identified only in the pulp extract. Methyl hexadecanoate was also previously reported in *Z. lotus* fruit essential oil, while methyl (9*Z*)-octadec-9-enoate is reported here for the first time as a component of *Z. lotus*. FAEEs and FAMEs have been reported to naturally occur in different plants and microalgae [28,29]. In fact, the presence of FAEEs and FAMEs in *Z. lotus* was previously reported, namely, in the *Z. lotus* fruit and in its essential oil, although without quantification [14,30]. Due to their promising biological activities [31], the investigation and quantification of these components in natural sources have become important.

#### 2.2.2. Monoglycerides

Particular attention should be paid to the values observed for several monoglycerides detected in all morphological parts of *Z. lotus* (Table 1). This family was concentrated in the leaves and seeds (189 and 255 mg kg^−1^ dw, respectively) due to the presence of 1-linolenoylglycerol in leaves and 1-oleoylglycerol in the seed extract, representing 44.4% and 60.8% of the total monoglyceride content, respectively. To our knowledge, the six monoglycerides are described here for the first time as components of *Z. lotus*.

#### 2.2.3. Long-Chain Aliphatic Alcohols

The profile of long-chain aliphatic alcohols (LCAAc) from the morphological parts of *Z. lotus* is reported here for the first time, as, in previous studies, only octacosanol has been identified in *Z. lotus* fruit (without quantification) [14]. Trace LCAAc accounted for 11.9% of the total amount of detected compounds (Table 1). This family is mainly concentrated in the pulp (340 mg kg^−1^ dw) and leaf extracts (438 mg kg^−1^ dw) (Figure 2), and octacosan-1-ol remains the predominant LCAAc (11–230 mg kg^−1^ dw), being present in all *Z. lotus* fractions, except in the seeds. Other LCAAc from C_14_ (tetradecan-1-ol) to C_30_ (triacontan-1-ol) were detected in root bark, leaf, and pulp extracts of *Z. lotus*, whereas, in the seeds, hexadecan-1-ol (2 mg kg^−1^ dw) was the LCAAc found.

#### 2.2.4. Pentacyclic Triterpenic Compounds

**Figure 3:**
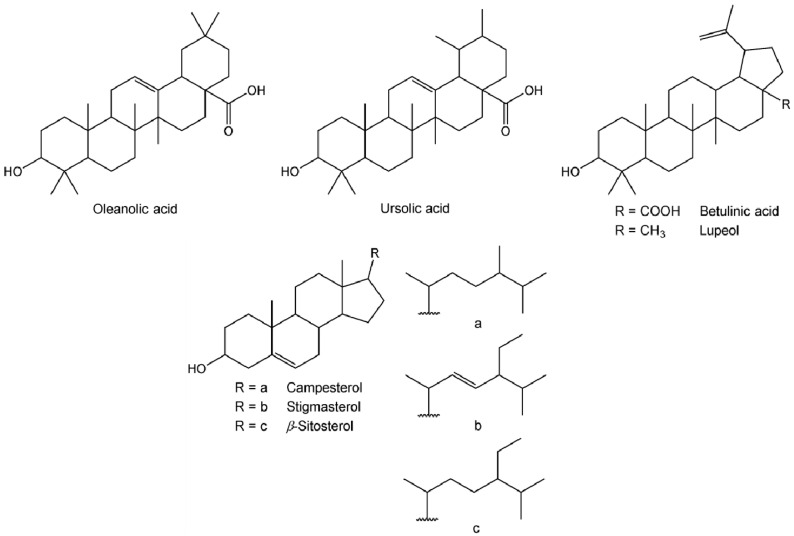
Structures of pentacyclic triterpenic compounds and sterols identified in *Z. lotus*.

Pentacyclic triterpenic compounds were the most abundant family of lipophilic compounds detected in root bark (Figure 2), accounting for 10,230 mg kg^−1^ dw. Their chemical structures are presented in Figure 3. Considerable amounts of triterpenes were also observed in the other morphological parts of *Z. lotus*, ranging from 248 mg kg^−1^ dw in leaves to 608 mg kg^−1^ dw in pulp. Betulinic acid (BA) was the major compound identified in root bark, accounting for 9838 mg kg^−1^ dw (492 mg g^−1^ of extract), which corresponds to 89.3% of the total lipophilic compounds identified in this extract. Given the broad range of pharmacological activities already known for BA [32], its abundance in root bark highlights the value of this shrub species as a promising source of high-value ingredients for nutraceutical and pharmaceutical applications. In fact, the BA concentration in *Z. lotus* L. root bark is in the range of that found in other natural rich sources, namely, the outer bark of *Eucalyptus nitens* (Myrtaceae) (6621 mg kg^−1^ dw) [33] and the bark of *Betula platyphylla suk.* (Betulaceae) (10,800 mg kg^−1^ dw) [32], using the same extraction solvent. Additionally, the presented BA content is 2.9-fold higher than in *Betula pendula* bark and up to 3.5-fold lower than in *Platanus acerifolia* (Platanaceae) cork [34]. Considerable amounts of BA were also observed in the other morphological parts of *Z. lotus*. In addition, all of the studied extracts were shown to be composed of oleanolic acid (OA), with contents from 51 mg kg^−1^ dw in leaves to 287 mg kg^−1^ dw in root bark. Lupeol was also identified in leaves and root bark, while ursolic acid was exclusively detected in the *Z. lotus* fruit, namely, in pulp and seeds.

Lupeol, oleanolic, betulinic and ursolic acids (Figure 3) are common triterpenic compounds in other *Zizyphus* species [35,36]; however, as far as our literature survey could ascertain, lupeol, betulinic and ursolic acids are identified for the first time as constituents of *Z. lotus*, while oleanolic acid has been previously identified in *Z. lotus* L. pulp [19].

#### 2.2.5. Sterols

Three sterols, the chemical structures of which are present in Figure 3, were also detected in *Z. lotus* extracts, particularly in root bark and leaves, with total contents of 257 and 355 mg kg^−1^ dw, respectively (Table 1). Beta-sitosterol was the main sterol observed in all morphological parts of *Z. lotus*, ranging from 68 mg kg^−1^ in pulp to 208 mg kg^−1^ in leaves. Stigmasterol was also found in pulp, leaves and root bark, while campesterol was only detected in root bark and leaves and was present in small amounts (4 and 28 mg kg^−1^ dw, respectively). To the best of our knowledge, this is the first study reporting the sterol profile of different *Z. lotus* morphological parts, although these compounds have been previously reported in *Z. lotus* fruit [14] and *Z. lotus* seed oil [12]. The presence of these sterols, particularly known for their various beneficial health effects [37] as *Z. lotus* components, increase the value of this shrub as a promising source of bioactive compounds.

#### 2.2.6. Aromatic and Other Compounds

Apart from the major families reported above, aromatic compounds are represented by 12 compounds, which were unequally distributed in the four morphological parts of *Z. lotus* in quite low amounts, ranging from 11 mg kg^−1^ dw in the root bark to 31 mg kg^−1^ dw in the seed extract (Table 1). Benzoic acid is the major aromatic compound detected in the pulp, while vanillin was mainly observed in the seed extract. Of all of the aromatic compounds detected, only benzoic and *p*-coumaric acids have been previously identified as components of *Z. lotus* fruits [14].

Finally, leaf extract revealed the presence of other minor but still interesting compounds, which distinguishes it from the remaining studied fractions (Table 1). Three positional isomers of neophytadiene were identified, as well as phytol, tetracosyl acetate, inositol and squalene, which were also found in significant amounts in the seed extract. Two long-chain aliphatic aldehydes were detected in pulp extracts, namely, octacosanal (52 mg kg^−1^ dw) and triacontanal (27 mg kg^−1^ dw), which were also previously detected in *Z. lotus* fruits, although their contents remained unknown [14].

Two isomers of vitamin E, namely, α-tocopherol and *γ*-tocopherol, were detected in the lipophilic leaf extract with a total amount of 128 mg kg^−1^ dw (Table 1). The contents of α-tocopherols in the four morphological parts of *Z. lotus* were mentioned in a previous report [4].

Other minor components, such as loliolide and solerol, were also detected in leaf and pulp extracts, respectively.

### 2.3. Biological Activities of Lipophilic Z. lotus Extracts

#### 2.3.1. Antiproliferative Activity

The antiproliferative effect of lipophilic extracts of different morphological parts of *Z. lotus*, as shown in Table 2, was evaluated for the first time against the MDA-MB-231 cell line. Cell viability was estimated by 3-(4,5-dimethylthiazol-2-yl)-2,5-diphenyltetrazolium bromide (MTT) assay. Lipophilic root bark extract showed a significant antiproliferative effect (half-maximal inhibitory concentration (IC_50_) of 4.23 µg mL^−1^) in vitro against the triple-negative breast cancer (TNBC) MDA-MB-231 cell line (Table 2), whereas lipophilic extracts of pulp, seeds and leaves had a lower inhibitory activity (IC_50_ higher than 50 µg mL^−1^ at 48 h) even compared with the IC_50_ reported for the ethanolic extract of Tunisian *Z. lotus* leaves (45.5 µg mL^−1^) [38].

The strong antiproliferative effect of lipophilic root bark extract is clearly related to its particular composition, as root bark revealed the highest content of lipophilic compounds, particularly pentacyclic triterpenic compounds (and BA in particular), which represent 10,230 mg kg^−1^ dw, corresponding to 92.9% of the total detected compounds (Table 1). In fact, these compounds can be responsible for the suppression of MDA-MB-231 cellular growth, and BA, which is the major compound of root bark extract (9838 mg kg^−1^ dw, 89.3% of the total detected compounds), is known to be a promising agent against different cancer types [39]. Several reports have demonstrated the efficacy of BA against TNBC, in which it causes cell cycle arrest and, ultimately, apoptosis [40,41]. Considering the BA concentration in root bark extract (491.9 mg g^−1^ extract), the determined IC_50_ value would be 2.1 µg of BA mL^−1^, which is clearly lower than the toxicity described in the literature for BA (IC_50_ within the 10–31 µg mL^−1^ concentration range) [39,41], suggesting the presence of a synergistic interaction between BA and other components of the extract, namely, oleanolic acid. In fact, BA, together with lupeol, was previously suggested to be responsible for the significant antiproliferative activity of a DCM bark extract from *Ziziphus mauritiana* against MCF-7 cells (IC_50_ = 5 µg mL^−1^) [42]. A synthetic mixture of BA plus OA in the same proportion as that in the root bark extract (97.1%BA:2.9%OA) presented a lower antiproliferative effect (IC_50_ of 15.27 µg mL^−1^) in the studied MDA-MB-231 cell line, underlining the synergetic contributions of other presented compounds in addition to OA.

Sterols are another family of bioactive compounds characterized by their anticancer potential [37]. Beta-sitosterol was found to inhibit MDA-MB-231 cell growth by inducing cell cycle arrest at the G2/M phase and as an anti-metastatic agent [43], while stigmasterol has antiproliferative activity, in contrast to cholesterol and campesterol, which were found to have no cytotoxic effect on MDA-MB-231 cell growth [44]. Considering the significant content of sterols in leaves and root bark (355 and 257 mg kg^−1^ dw, respectively), these compounds could also be involved in the obtained results. Therefore, a more in-depth analysis is necessary to better understand the suppressing effect of lipophilic root bark extracts on MDA-MB-231 cell viability and the possible synergistic actions between extract components.

#### 2.3.2. Antibacterial Activity

The antibacterial activity of the different lipophilic extracts of *Z. lotus* was evaluated against the bacterial strains *E. coli*, MSSA and *S. epidermidis*. The results obtained using the Resazurin assay (Table 3) demonstrated that the leaf extract exerted the highest activity among all fractions, as it had an inhibitory effect on all strains studied, especially for *E. coli* and *S. epidermidis*, with a minimum inhibitory concentration (MIC) of 1024 µg mL^−1^. In contrast, lipophilic pulp extract did not demonstrate antibacterial activity against any of the studied bacterial strains in the concentration range used. Moreover, the lipophilic extracts of leaves and root bark showed a slightly inhibitory effect on MSSA (MIC of 2048 μg mL^−1^). *S. epidermidis* showed susceptibility to *Z. lotus* extracts between 1024 (for seed and leaf extracts) and 2048 µg mL^−1^ (for root bark extract).

According to the literature, our results revealed that *Z. lotus* leaf extract had a stronger effect on *E. coli* when compared to the leaf methanolic extract analyzed by Ghazghazi et al. [13] (MIC of 12,500 µg mL^−1^) but similar to the methanolic extracts of *Z. lotus* studied by Naili et al. (2010) [45] (MIC of 1000 µg mL^−1^). Moreover, Ghazghazi et al. (2014) [13] also studied anti-*S. aureus* activity, which was shown to be less effective (MIC of 25,000 µg mL^−1^). However, other *Z. lotus* leaf extracts showed higher antibacterial activities, such as the acetone-derived extracts reported by Tlili et al. [46], which presented MIC values of 1000 µg mL^−1^ and 250 µg mL^−1^ against MSSA and *S. epidermidis* ATCC 35984, respectively, as well as the aqueous extract of leaves obtained by Rached et al. [8], which was shown to be effective against MSSA (MIC of 1250 µg mL^−1^).

UFAs were identified as the major components of the lipophilic extract of *Z. lotus* leaves, which suggests that they may be responsible for the antibacterial activity of this extract. In fact, these components are known to have promising antibacterial activities against both Gram-positive and Gram-negative bacteria by destabilizing bacterial cell membranes [47]. However, a significantly higher content of UFA was detected in seed extract, which only presented antibacterial capacity against *S. epidermidis*, and in an amount similar to that in leaf extract. This highlights that other minor components can be responsible or promote synergisms, leading to the reported leaf extract’s antibacterial activity. This can be attributed to long-chain aliphatic alcohols, which have been described as potent antibacterial agents [48,49].

In contrast, the antibacterial activity of root bark against MSSA and *S. epidermidis* may be attributed to their major compounds, namely, pentacyclic triterpenic compounds, particularly BA. This lipophilic compound is known for having antibacterial activity against *Staphylococcus* spp. (MIC of 64 µg mL^−1^ against MSSA), with evidence indicating that the cell membrane is the main target via interference with peptidoglycan biosynthesis [50].

Based on our findings, lipophilic *Z. lotus* extracts, particularly root bark and leaves, show interesting biological potential due to their favorable chemical composition. *Z. lotus* is a source of valuable bioactive compounds, and as a widespread indigenous plant occurring in arid and semiarid plateau regions of North Africa, this plant can integrate additional economic valorization with health-promoting solutions, such as human nutrition or pharmaceutics.

## 3. Materials and Methods

### 3.1. Reagents

Nonadecan-1-ol (99% purity), hexadecanoic acid (≥99% purity), *β*-stigmasterol (95% purity), vanillin (99%), anhydrous pyridine (99.8% purity), dichloromethane (99% purity), *N*,*O*-bis(trimethylsilyl)trifluoroacetamide (99% purity), trimethylchlorosilane (99% purity) and tetracosane (99% purity) were obtained from Sigma Chemicals Co. (Madrid, Spain). Ursolic acid (98% purity) was purchased from Aktin Chemicals (Chengdu, China). Dimethyl sulfoxide (DMSO), cell culture grade, was obtained from PanReac Applichem (Gatersleben, Germany). Acetone (≥99% purity) was supplied by VWR (Radnor, OA, USA). 3-(4,5-Dimethylthiazol-2-yl)-2,5-diphenyltetrazolium bromide was obtained from Calbiochem (San Diego, CA, USA), and Mueller–Hinton agar or broth was purchased from Liofilchem (Roseto degli Abruzzi, Italy). Brucella Broth was purchased from Fluka Analytical, and Resazurin was obtained from Sigma-Aldrich (St. Louis, MI, USA).

### 3.2. Samples

Wild *Z. lotus* was collected from the regions of Beni Mellal, Morocco (32°20′21.998″ N; 6°21′38.999″ W), between September and October 2016. Species identification was performed based on the botanical criteria of the authors and authenticated by Professors of Botany at the University of Sultan Moulay Slimane, Morocco. The shrub was separated manually into four different morphological parts, namely, root bark, leaves, pulp and seeds; each fraction was shade-dried (15 days) and milled to granularity lower than 2 mm prior to extraction.

### 3.3. Extraction

Adequate mass (15 g of dw) from each *Z. lotus* part, i.e., root bark, leaves, pulp and seeds, were Soxhlet-extracted with DCM (150 mL) for 8 h in order to obtain the lipophilic extractives. DCM was chosen since it is known to be a specific solvent to extract lipophilic compounds from plants [51]. The solvent was evaporated to dryness under vacuum using a rotary evaporator, and the extracts were weighed. Extractions were performed in triplicate, and the results are expressed as percentage of dw material (% *w*/*w*).

### 3.4. GC–MS Analysis

#### 3.4.1. Derivatization

Before GC–MS analysis, approximately 20 mg of each dried DCM extract was dissolved in 250 μL of pyridine containing 1 mg of tetracosane used as an internal standard. The compounds with carboxylic and hydroxyl groups were converted into their trimethylsilyl derivatives by adding 250 µL of *N*,*O*-bis-(trimethylsilyl)trifluoroacetamide, 50 µL of trimethylchlorosilane and 250 µL of pyridine. The mixture was heated at 70 °C for 30 min. The trimethylsilyl derivatives were analyzed by GC–MS.

#### 3.4.2. GC–MS Conditions

GC–MS-QP2010 Ultra (Shimadzu, Kyoto, Japan) was used to analyze the derivatized extracts. A DB-1 J&W capillary column (30 m × 0.32 mm i.d., 0.25 μm film thickness, Santa Clara, CA, USA) was used to separate lipophilic compounds, using helium (at a flow rate of 35 cm s^−1^) as the carrier gas. The chromatographic conditions were as follows: initial temperature, 80 °C for 5 min; first temperature gradient, 4 °C min^−1^ up to 260 °C; second temperature gradient, 2 °C min^−1^ up to 285 °C for 8 min; injector temperature, 250 °C; transfer-line temperature, 290 °C; split ratio, 1:50. The injected volume was 1 µL.

Lipophilic compounds were identified by comparing their mass spectra with the GC–MS spectral libraries (Wiley 275 and U.S. National Institute of Science and Technology (NIST14)), with their retention time obtained under the same conditions [52,53,54], and comparing their MS fragmentation profiles with the literature [55,56,57,58,59,60].

#### 3.4.3. Quantitative Analysis

Lipophilic compounds were quantified by their peak areas, with GC–MS being calibrated with pure reference compounds (after derivatization as described above) representative of each family, namely, hexadecanoic acid, nonadecan-1-ol, vanillin, *β*-stigmasterol and ursolic acid, relative to tetracosane (the internal standard). The respective response factors were calculated as the average of six GC−MS runs, with less than 5% variation between injections. The triplicates of each lipophilic extract were injected in duplicate, and the results, expressed in milligrams per kilogram of dw, represent the average of the concordant values obtained for the six runs.

### 3.5. Antiproliferative Activity

#### 3.5.1. Cell Culture

The human triple-negative breast cancer (TNBC) MDA-MB-231 cell line was obtained from American Type Cell Culture (Manassas, VA, USA). MDA-MB-231 cells were cultured in Dulbecco’s Modified Eagle Medium (DMEM) (Biowest, Nuaillé, France) supplemented with 10% (*v*/*v*) heat-inactivated fetal bovine serum (FBSi) (Sigma-Aldrich, St. Louis, MI, USA) and 1% penicillin–streptomycin mixture (Biowest, Nuaillé, France). The cells were maintained at 37 °C in a 5% CO_2_ humidified atmosphere (C150, Binder GmbH, Tuttlingen, Germany). Before confluence, the cells were washed with phosphate-buffered saline (PBS), harvested with the addition of a trypsin solution (0.5 gL^−1^)/EDTA (0.2 gL^−1^) (Biowest, Nuaillé, France) and suspended in fresh growth medium before plating. All experiments were performed during the linear phase of cellular growth.

#### 3.5.2. Cell Viability Assay

MDA-MB-231 cells were seeded in 96-well plates at 2 × 10^5^ cells/mL and allowed to adhere for 24 h at 37 °C. Cells were then incubated with different lipophilic extracts of *Z. lotus* (root bark, leaves, seeds and pulp) at different concentrations (0.1, 5, 10, 20, 50 and 100 μg mL^−1^) for 48 h. Under the same conditions, cells were also incubated with a synthetic root bark mixture in the proportion of 2.9% OA plus 97.1% BA. Vehicle solvent control cells received dimethyl sulfoxide (DMSO) (<1% (*v*/*v*)), cell culture grade (Applichem, Germany). Cell viability was estimated by 3-(4,5-dimethylthiazol-2-yl)-2,5-diphenyltetrazolium bromide (MTT) (Calbiochem, San Diego, CA, USA) assay as previously described [61]. Briefly, 20 μL of MTT stock solution was added to each well (final concentration 0.5 mg mL^−1^), followed by an incubation period of 4 h. A DMSO/ethanol (1:1) solution was then added to dissolve the formed formazan crystals, followed by a spectrophotometric determination at 570 nm (MultiSkan FC, Thermo Scientific, Rochester, NY, USA). Results are expressed as the percentage of cell viability relative to the control (cells with vehicle solvent). IC_50_, defined as the concentration necessary to cause 50% inhibition of cell viability, was calculated using GraphPad Prism 5.0 (GraphPad Prism Software Inc., San Diego, CA, USA) by plotting the percentage of cell viability as a function of sample concentration logarithm. Triplicates were performed in three independent experiments for each treatment.

#### 3.5.3. Statistical Analysis

The statistical treatment of the antiproliferative activity data was carried out through one-way analysis of variance (ANOVA) using IBM^®^ SPSS ^®^ Statistics version 25 (IBM Corporation, New York, NY, USA). The source of the differences was identified through the Student’s *t*-test with *p* < 0.05 as the significance level.

### 3.6. Antibacterial Activity

The antibacterial activity of lipophilic *Z. lotus* extracts (root bark, leaves, pulp and seeds) was determined using the minimal inhibitory concentration (MIC) through the microbroth dilution method. Extracts were tested against the bacterial strains *E. coli* ATCC 25922, *S. aureus* ATCC 6538 (methicillin-sensitive *Staphylococcus aureus*—MSSA) and *S. epidermis* (clinical isolate), which were kindly provided by Portuguese Catholic University (Porto, Portugal). These bacterial strains were maintained at −80 °C in Brucella Broth supplemented with 5% DMSO until use. Assays were performed with bacterial cultures grown in Mueller–Hinton agar (MHA) plates incubated overnight. Briefly, bacterial strains in the exponential growth phase were suspended in Mueller–Hinton broth (MHB) to obtain a final inoculum concentration of 1 × 10^5^ CFU mL^−1^ according to Clinical and Laboratory Standards Institute guidelines [62].

Serial dilutions of lipophilic *Z. lotus* extracts in 96-well plates were performed using concentrations between 8 and 2048 μg mL^−1^. The following controls were also performed: (i) solvent control: bacterial cultures with 4% (*v*/*v*) DMSO or acetone; (ii) growth control: bacterial inoculum; and (iii) sterility control: culture media. Three independent experiments were performed for each extract, each one in triplicate. The MIC values were determined after 24 h of incubation at 37 °C by using the Resazurin assay adapted from Sarker et al. (2007) [63]. MIC was considered to be the minimum concentration of the tested sample at which the color did not change from blue to pink and did not fluoresce when reduced to resorufin by oxidoreductases within viable cells.

## 4. Conclusions

The lipophilic fractions of *Z. lotus* pulp, seeds, leaves and root bark were characterized in detail by gas chromatography–mass spectrometry, allowing the identification and quantification of 99 compounds, including fatty acids, long-chain aliphatic alcohols, pentacyclic triterpenic compounds, sterols, monoglycerides, aromatic compounds and other minor components. The four studied morphological parts of *Z. lotus* were determined to be composed of valuable bioactive lipophilic compounds. Root bark, in particular, was observed to be a source of betulinic acid (9838 mg kg^−1^ dw). The extract of this morphological part showed promising antiproliferative activity against a triple-negative breast cancer cell line, MDA-MB-231, while leaf extract revealed interesting antimicrobial activity against *Escherichia coli*, methicillin-sensitive *Staphylococcus aureus* and *Staphylococcus epidermis*. This study highlights the potential of *Zizyphus lotus* L., promoting its economic exploitation as a natural ingredient in pharmaceutical formulations, which can only be implemented after the development of sustainable extraction methodologies, a careful evaluation of technical and economic aspects and, finally, an analysis to ensure that their exploitation does not have an ecological impact. Ultimately, this means that the sustainable exploitation of the plant (integrated with fruit exploitation) or, ultimately, its cultivation as a dedicated crop can also be considered. Thus, this study represents an important step by identifying the potential of this plant as a source of bioactive compounds, but further studies encompassing the sustainable exploitation of the plant will be necessary.

## Figures and Tables

**Table 1:** Quantitative analysis (mg kg^−1^ of dry weight) of lipophilic extracts derived from four morphological parts of *Zizyphus lotus* L.

RT (min)	Compound	Pulp	Seeds	Leaves	Root Bark
Fatty acids		1469	8512	4643	434
*Saturated fatty acids*		799	1260	1461	265
Decanoic acid	19.50	67	5	5	1
Undecanoic acid	22.58	9	n.d.	n.d.	n.d.
Dodecanoic acid	25.49	17	2	4	2
Tridecanoic acid	28.25	4	n.d.	n.d.	n.d.
Tetradecanoic acid	30.89	28	10	52	n.d.
Pentadecanoic acid	33.40	9	4	5	2
Hexadecanoic acid	35.82	366	594	877	152
Heptadecanoic acid	38.10	22	7	8	7
Octadecanoic acid	40.32	42	570	276	49
Nonadecanoic acid	42.43	2	n.d.	n.d.	1
Eicosanoic acid	44.48	16	48	40	7
Heneicosanoic acid	46.48	5	n.d.	3	3
Docosanoic acid	48.34	10	19	25	13
Tricosanoic acid	50.16	n.d.	n.d.	n.d.	9
Tetracosanoic acid	52.04	10	n.d.	26	14
Pentacosanoic acid	54.02	3	n.d.	9	5
Hexacosanoic acid	56.10	10	n.d.	52	n.d.
Heptacosanoic acid	58.30	11	n.d.	n.d.	n.d.
Octacosanoic acid	60.56	114	n.d.	79	n.d.
Triacontanoic acid	65.51	54	n.d.	tr	n.d.
*Unsaturated fatty acids*		421	7222	3175	159
Tetradecenoic acid	30.19	2	n.d.	n.d.	n.d.
Hexadecenoic acid isomer a	35.05	4	10	4	2
Hexadecenoic acid isomer b	35.19	40	11	23	2
Hexadecenoic acid isomer c	35.44	7	n.d.	n.d.	2
Heptadecenoic acid isomer a	37.44	24	7	n.d.	n.d.
Heptadecenoic acid isomer b	37.52	n.d.	n.d.	n.d.	1
Heptadecenoic acid isomer c	37.60	n.d.	n.d.	n.d.	1
(9*Z*,12*Z*)-Octadeca-9,12-dienoic acid	39.42	60	737	544	50
(9*Z*,12*Z*,15*Z*)-Octadeca-9,12,15-trienoic acid	39.50	45	n.d.	2431	9
(9*Z*)-Octadec-9-enoic acid	39.62	179	6255	120	59
(9*E*)-Octadec-9-enoic acid	39.78	50	135	54	18
Nonadecenoic acid	41.72	7	n.d.	n.d.	n.d.
Eicos-11-enoic acid	43.83	2	66	<0.5	14
*Diacids*		7	n.d.	n.d.	n.d.
Hexadecanedioic acid	45.19	7	n.d.	n.d.	n.d.
*ω-Hydroxy fatty acids*		10	n.d.	7	9
22-Hydroxydocosanoic acid	55.04	5	n.d.	7	9
2-Hydroxytetracosanoic acid	55.24	5	n.d.	n.d.	n.d.
*Fatty acid ethyl esters*		228	16	n.d.	n.d.
Ethyl decanoate	17.08	1	n.d.	n.d.	n.d.
Ethyl tetradecanoate	29.11	11	n.d.	n.d.	n.d.
Ethyl pentadecanoate	31.74	4	n.d.	n.d.	n.d.
Ethyl hexadec-9-enoate isomer a	33.62	12	n.d.	n.d.	n.d.
Ethyl hexadec-9-enoate isomer b	33.86	3	n.d.	n.d.	n.d.
Ethyl hexadecanoate	34.26	104	5	n.d.	n.d.
Ethyl (9*Z*)-octadec-9-enoate	38.24	33	11	n.d.	n.d.
Ethyl (9*E*)-octadec-9-enoate	38.39	25	n.d.	n.d.	n.d.
Ethyl octadecanoate	38.95	29	n.d.	n.d.	n.d.
Ethyl eicosanoate	43.27	6	n.d.	n.d.	n.d.
*Fatty acid methyl esters*		5	15	n.d.	1
Methyl hexadecanoate	32.53	5	n.d.	n.d.	n.d.
Methyl (9*Z*)-octadec-9-enoate	36.67	n.d.	15	n.d.	1
Monoglycerides		27	255	189	24
2-Palmitoylglycerol	47.05	n.d.	3	5	n.d.
1-Palmitoylglycerol	47.67	13	44	47	12
1-Linoleoylglycerol	50.61	n.d.	35	30	3
1-Linolenoylglycerol	50.72	n.d.	n.d.	84	n.d.
1-Oleoylglycerol	50.73	14	155	n.d.	4
1-Stearoylglycerol	51.28	n.d.	17	24	4
Long chain aliphatic alcohols		340	2	438	51
Tetradecan-1-ol	28.89	n.d.	n.d.	n.d.	2
Hexadecan-1-ol	33.96	4	2	4	9
(9*Z*)-Octadec-9-en-1-ol	37.87	9	n.d.	11	14
Octadecan-1-ol	38.60	4	n.d.	2	6
Docosan-1-ol	46.83	3	n.d.	n.d.	3
Tetracosan-1-ol	50.51	n.d.	n.d.	5	3
Hexacosan-1-ol	54.36	7	n.d.	118	3
Heptacosan-1-ol	56.46	8	n.d.	23	n.d.
Octacosan-1-ol	58.65	207	n.d.	230	11
Nonacosan-1-ol	60.91	19	n.d.	13	n.d.
Triacontan-1-ol	63.24	79	n.d.	31	n.d.
Pentacyclic triterpenic compounds		608	483	248	10230
Lupeol	63.86	n.d.	n.d.	78	105
Oleanolic acid	69.21	103	164	51	287
Betulinic acid	69.82	160	238	119	9838
Ursolic acid	70.72	345	81	n.d.	n.d.
Sterols		81	96	355	257
Campesterol	60.70	n.d.	n.d.	28	4
Stigmasterol	61.41	13	n.d.	119	126
*β*-Sitosterol	62.79	68	96	208	127
Aromatic compounds		29	31	21	11
Benzoic acid	11.71	23	2	5	n.d.
Vanillin	21.00	n.d.	20	n.d.	3
Salicylic acid	21.02	n.d.	n.d.	4	n.d.
Vanillyl alcohol	24.99	n.d.	5	n.d.	1
Syringaldehyde	25.93	n.d.	n.d.	n.d.	1
Homovanillyl alcohol	27.00	n.d.	n.d.	n.d.	2
Vanillic acid	28.39	4	4	n.d.	2
Hydroxytyrosol	28.96	n.d.	n.d.	n.d.	2
Protocatechuic acid	30.42	n.d.	n.d.	n.d.	<0.5
Syringic acid	31.86	n.d.	n.d.	n.d.	1
*p*-Coumaric acid	32.88	2	n.d.	5	n.d.
*E*-Ferulic acid	36.54	n.d.	n.d.	7	n.d.
Others		151	228	832	9
Solerol	13.45	6	n.d.	n.d.	n.d.
Glycerol	14.21	41	148	251	9
Loliolide	28.26	n.d.	n.d.	39	n.d.
Neophytadiene isomer a	30.76	tr	n.d.	141	n.d.
Neophytadiene isomer b	31.29	n.d.	n.d.	29	n.d.
Neophytadiene isomer c	31.75	n.d.	n.d.	49	n.d.
Inositol	36.94	n.d.	n.d.	13	n.d.
Phytol	39.04	n.d.	n.d.	117	n.d.
Squalene	51.53	n.d.	79	39	n.d.
*γ*-Tocopherol	55.21	n.d.	n.d.	54	n.d.
Tetracosyl acetate	55.33	n.d.	n.d.	26	n.d.
Octacosanal	55.82	52	n.d.	n.d.	n.d.
Nonacosan-10-one	56.86	24	n.d.	n.d.	n.d.
*α*-Tocopherol	58.19	n.d.	n.d.	74	n.d.
Triacontanal	60.35	27	n.d.	n.d.	n.d.
**Total**		**2704**	**9607**	**6726**	**11016**

Results represent the average of the concordant values obtained for six aliquots of each sample, with less than 5% variation between samples. Abbreviations: n.d. not detected; tr, traces.

**Table 2:** IC50 values of lipophilic extracts of *Z. lotus* on TNBC cell line MDA-MB-231, obtained by using the MTT assay.

Lipophilic *Z. lotus* Extract	MDA-MB-231 (IC_50_ µg mL^−1^)
Pulp	>50
Seeds	>50
Leaves	>50
Root bark	4.23 ± 0.18 ^a^
Synthetic root bark mixture *	15.27 ± 1.79 ^b^

Data are expressed as mean ± standard error (n = 3). Means with different letters are statistically different at *p* < 0.05 using Student’s *t*-test. * BA + OA (97.1%:2.9%).

**Table 3:** MIC values of lipophilic *Z. lotus* extracts against *E. coli*, MSSA and *S. epidermidis*, determined through Resazurin assay.

*Z. lotus* Extract	MIC (μg mL^−1^)		
	*E. coli*	Methicillin-Sensitive *Staphylococcus aureus* (MSSA)	*S. epidermidis*
Pulp	>2048	>2048	>2048
Seeds	>2048	>2048	1024
Leaves	1024	2048	1024
Root bark	>2048	2048	2048

## Data Availability

Not applicable.

## Sample Availability

Samples of the compounds are not available from the authors.
